# Bronchoscopic assessment of airway retention time of aerosolized xylitol

**DOI:** 10.1186/1465-9921-7-27

**Published:** 2006-02-16

**Authors:** Lakshmi Durairaj, Srividya Neelakantan, Janice Launspach, Janet L Watt, Margaret M Allaman, William R Kearney, Peter Veng-Pedersen, Joseph Zabner

**Affiliations:** 1 Department of Medicine, Roy J. and Lucille A. Carver College of Medicine, University of Iowa, Iowa City, IA, USA; 2 Division of Pharmaceutics, College of Pharmacy, University of Iowa, Iowa City, IA, USA

## Abstract

**Background:**

Human airway surface liquid (ASL) has abundant antimicrobial peptides whose potency increases as the salt concentration decreases. Xylitol is a 5-carbon sugar that has the ability to lower ASL salt concentration, potentially enhancing innate immunity. Xylitol was detected for 8 hours in the ASL after application in airway epithelium *in vitro*. We tested the airway retention time of aerosolized iso-osmotic xylitol in healthy volunteers.

**Methods:**

After a screening spirometry, volunteers received 10 ml of nebulized 5% xylitol. Bronchoscopy was done at 20 minutes (n = 6), 90 minutes (n = 6), and 3 hours (n = 5) after nebulization and ASL was collected using microsampling probes, followed by bronchoalveolar lavage (BAL). Xylitol concentration was measured by nuclear magnetic resonance spectroscopy and corrected for dilution using urea concentration.

**Results:**

All subjects tolerated nebulization and bronchoscopy well. Mean ASL volume recovered from the probes was 49 ± 23 μl. The mean ASL xylitol concentration at 20, 90, and 180 minutes was 1.6 ± 1.9 μg/μl, 0.6 ± 0.6 μg/μl, and 0.1 ± 0.1 μg/μl, respectively. Corresponding BAL concentration corrected for dilution was consistently lower at all time points. The terminal half-life of aerosolized xylitol obtained by the probes was 45 minutes with a mean residence time of 65 minutes in ASL. Corresponding BAL values were 36 and 50 minutes, respectively.

**Conclusion:**

After a single dose nebulization, xylitol was detected in ASL for 3 hours, which was shorter than our *in vitro *measurement. The microsampling probe performed superior to BAL when sampling bronchial ASL.

## Introduction

Human airway surface liquid (ASL) contains many antimicrobial substances, including lysozyme, lactoferrin, and β defensins that are salt-sensitive. An increase in salt concentration inhibits the antibacterial activity of these substances. Conversely, they are more potent at lower salt concentrations [1-4].

Xylitol is a five-carbon sugar that is used as a nutritive sweetener. When added to the apical surface of airway epithelia, it can lower the ASL salt concentration, resulting in enhanced antimicrobial properties. Using a radiotracer method, we found that xylitol has low permeability across an *in vitro *model of well-differentiated human airway epithelia. Following addition to the apical surface, the amount of xylitol in the ASL decreased progressively; after 8–12 hours, only 50% of the applied sugar had diffused to the basolateral surface [5]. We recently tested the safety of aerosolized xylitol in normal volunteers. All subjects tolerated the exposures well without any significant change in Forced Expiratory Volume (FEV) 1, or laboratory parameters [6].

The main aim of this study is to assess the rate at which xylitol disappears from the ASL. It is difficult to measure the actual salt concentration in the ASL because collecting the fluid induces instantaneous changes in its composition [7]. Currently, the most widely used method for sampling ASL is bronchoalveolar lavage (BAL); however, BAL has limitations. First, it requires instillation of normal saline into lung segments, resulting in enormous dilution of the ASL. Second, there is a highly variable return of the instilled liquid. Third, the relative contribution of airway surface is insignificant compared to the alveolar surface sampled by BAL. This results in underrepresentation of the airway component when sampling ASL. Recently, a new method for sampling human airway surface liquid using a bronchoscopic microsampling (BMS) probe was reported by Ishizaka et al [8]. This method has been used to determine the ASL concentration of Levaquin after oral administration [9]. We describe the results of xylitol concentration in ASL obtained using a microsampling probe after aerosolization and compare it with the traditional BAL sampling.

## Methods

### Xylitol permeability in human airway epithelia *in vitro*

For an osmolyte to lower ASL salt concentrations, it must remain in ASL for some period of time before being absorbed or cleared by the airway epithelium. We tested the permeability of xylitol across well-differentiated airway epithelia using proton nuclear magnetic resonance (NMR) spectroscopy and compared it with the results from our previous experiment using ^14^C-labeled xylitol tracer [5]. Xylitol (8 μmol in 60 μl) was added to the apical surface of well-differentiated airway epithelia at time zero. Apical liquid was then removed at 2, 4, 6, and 8 hours, and the xylitol concentration quantitated by NMR spectroscopy.

### Healthy volunteer study

The study was approved by the University of Iowa Institutional Review Board and the Food and Drug Administration. After obtaining written informed consent, 18 subjects between the ages of 18 and 45 were consented. Exclusion criteria were FEV1 < 85% predicted, pregnancy, any chronic medical condition, or known allergy to lidocaine. Subjects received 10 ml of 5% xylitol (Danisco Cultor, Kansas). Aerosolization was generated using a Pari LC Plus nebulizer with Proneb Ultra compressor system (Pari Inc, Monterey, CA). Xylitol was prepared by mixing crystal sugar in sterile water (Abbott laboratories, IL) as previously described [6]. The first group of subjects (n = 6) underwent bronchoscopy 20 minutes after nebulization, the second group (n = 6) at 90 minutes, and the final group (n = 5) at 180 minutes. One subject recruited for the 180-minute group was excluded because of screening FEV1 < 85% predicted and wasn't replaced. Blood was drawn at baseline to obtain serum urea measurements.

### Bronchoscopy

All bronchoscopies were performed by the same staff physicians in a standard fashion using a flexible fiberoptic bronchoscope (model BF-30; Olympus). Subjects were given local anesthesia using 4% lidocaine sprayed in the oropharynx and 1% lidocaine directly instilled over the vocal cords, carina, and both the main stem bronchi. Under monitored sedation using intravenous midazolam and fentanyl, bronchoscope was introduced into the right lower lobe bronchus. After the channel was flushed with air, a microsampling probe (model BC-401C; Olympus Optical CO., LTD, Tokyo) was inserted into a segmental bronchus as previously described [9]. The microsampling probe has an outer sheath (1.8 mm diameter) and an inner probe (1.1 mm diameter and 3 cm length) attached to a stainless steel guide wire. The inner probe was advanced into a distal airway, positioned against the bronchial wall for 10 seconds to collect ASL, and then withdrawn into the outer sheath. The probe with the sheath was removed and the 3-cm tip of the inner probe was cut into a pre-weighed tube. This procedure was done three times and the sectioned inner probes were weighed. BAL was then performed by instilling two 20-ml aliquots of sterile normal saline into the lingula and right middle lobe as previously described [10].

### Specimen processing

BAL fluid was processed as previously described [6]. Briefly, the fluid was filtered through two layers of sterile gauze and centrifuged for 10 minutes at 1500 rpm. The cell-free fluid was frozen at -70°C until required for assays. Microsampling probes were added to 2 ml of saline in a pre-weighed tube, vortexed for 1 minute, and the solution was stored at -20°C. The probes were then dried and weighed again to measure the volume of ASL recovered.

### Nuclear magnetic resonance

NMR spectrometry is a form of absorption spectrometry where the absorption of radio waves by the nuclei of a molecule is a function of the structure of the molecule [11]. The equipment used is a proton spectrometer, which detects proton signals depending on the relative orientation of the hydrogen ions to the carbon moiety [500 mHz NMR system (Varian Inova 500, Varian Inc., Palo Alto, CA)]. NMR identifies xylitol and determines its concentration using area under the signal peak which indicates the number of protons of that type and hence concentration of the molecule. The NMR assay is very specific for xylitol given its unique structure (C_5_H_12_O_5_) and its range of detection is 5 μM – 6 M (0.0008 – 912 μg/μl). To assess for interference with lidocaine, we obtained an NMR spectrum of lidocaine and found that the signal peaks for lidocaine and xylitol did not overlap. (data not shown).

### Xylitol concentration

Xylitol concentration in the ASL was calculated using the previously described formula: ASL xylitol concentration = BMS xylitol concentration × (2 + ASL volume)/(ASL volume) [9]. ASL volume recovered by the probes was estimated by subtracting the wet and dry weight of the probes. Xylitol concentration in the BAL was adjusted for dilution using this formula: Alveolar concentration = (Concentration in BAL × serum urea)/BAL urea (Infinity urea assay kit, Thermo Electron, Melbourne, Australia). Urea nitrogen concentration in the BAL is usually identical to corresponding serum concentration, as urea readily crosses the alveolar-capillary membrane barrier [12].

### Pharmacokinetic and statistical analysis

Xylitol concentrations for both methods were more than two standard deviations higher for three subjects relative to the remaining subjects. Accordingly, these measurements were considered outliers and were therefore omitted from the analysis. These three subjects had similar baseline characteristics as the other subjects. Xylitol concentration in ASL obtained by the BAL and BMS methods were analyzed using the one-compartment model with first-order elimination and discontinuous zero-order input.



In Equation 1, c(t) is the xylitol concentration at time = t, R is the zero-order input rate constant, V is the volume of distribution, k is the elimination rate constant and T is the nebulization time. The term (t-T)_+ _is defined by:



Pharmacokinetic data from the remaining subjects were simultaneously fitted to obtain population values for k and R/V (volume-normalized input rate constant) parameters for different nebulization times using the interactive WINFUNFIT. WINFUNFIT is a Windows-based application, evolved from the general nonlinear regression program FUNFIT [13].

The mean residence time (MRT), that provides an estimate for the average time spent by xylitol molecules in the ASL, was calculated as the reciprocal of k. Mean time parameters may be generally defined as the average time for a kinetic event to occur [14,15], and the residence time of drug in a certain space qualifies as a kinetic event.

## Results

### Half-life of xylitol in airway epithelia

**Figure 1:**
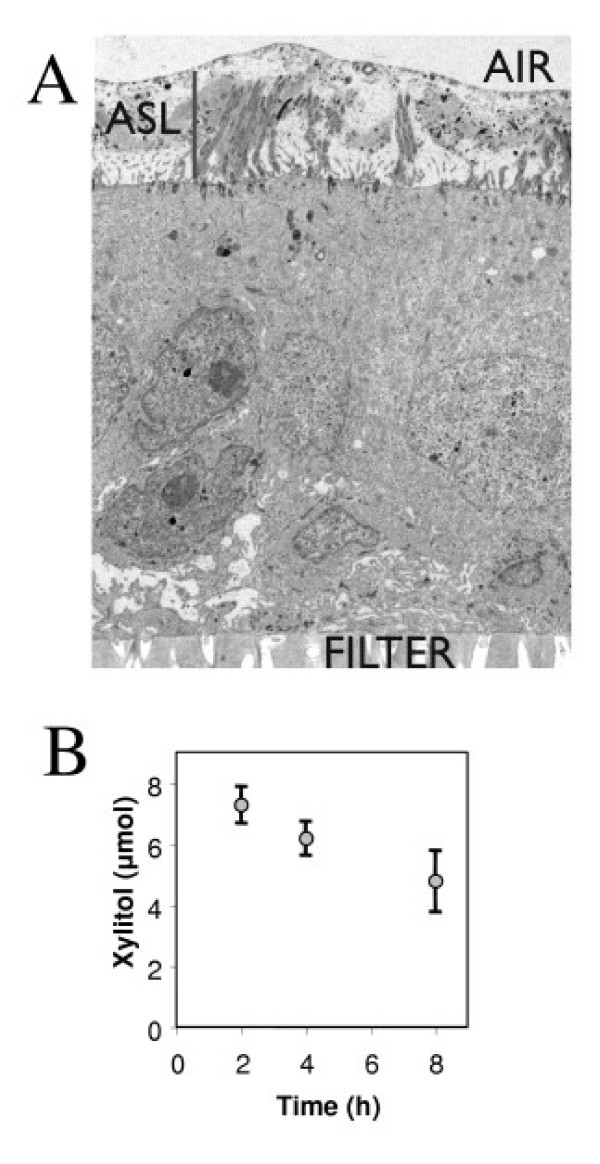
***In vitro *half-life of xylitol in human airway epithelia**. Panel A. Transmission electron micrograph of perfluorocarbon/OsO4 fixed human airway epithelia grown on a semi-permeable membrane filter. The vertical bar in the left upper quadrant shows ASL height which measured 5 μm. Panel B. Xylitol concentration in the basolateral surface quantitated by NMR after addition to the apical surface.

Following addition to the apical surface, the amount of xylitol in the ASL progressively decreased; after 8 hours, only 50% of the applied sugar had diffused to the basolateral surface (Figure 1). This low permeability suggests that xylitol could effectively hold liquid on the apical surface and lower the salt concentration. These results obtained using NMR measurements were very similar to those obtained using the radiotracer method, which was previously published [5].

### Human study

All subjects tolerated the xylitol nebulization and bronchoscopy well. Mean age of the subjects was 26 years (range 19–43), with a mean body mass index of 26.4 (standard deviation 2.8). All the subjects were nonsmokers. Current medications used by the subjects included contraceptive pills (n = 2), alprazolam and escitalopram (n = 1), and minocycline (n = 1). The average nebulization time of 10 mL xylitol using Pari-LC nebulizer was 48 ± 11 minutes.

**Figure 2:**
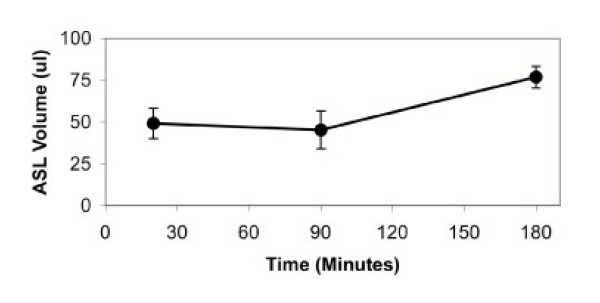
ASL volume recovered by BMS probe over time. * p = 0.03.

The mean ASL volume obtained using the microsampling probe was 56 ± 6 μl, and was higher at 180 minutes as compared to 20 minutes (77 ± 7 vs. 49 ± 9, p = 0.03, Figure 2). If ASL remains isotonic, these data suggest that xylitol both lowers the salt concentration and increases the ASL volume. Using urea dilution method, we estimated the volume of epithelial lining liquid sampled by BAL to be 16 ± 9 μl, 16 ± 11 μl, and 14 ± 7 μl at 20, 90, and 180 minutes, respectively. These volumes are lower than those measured by the BMS probe method. However, whereas the BMS probe method only samples bronchial surface liquid, BAL samples both bronchial and alveolar liquid.

**Figure 3:**
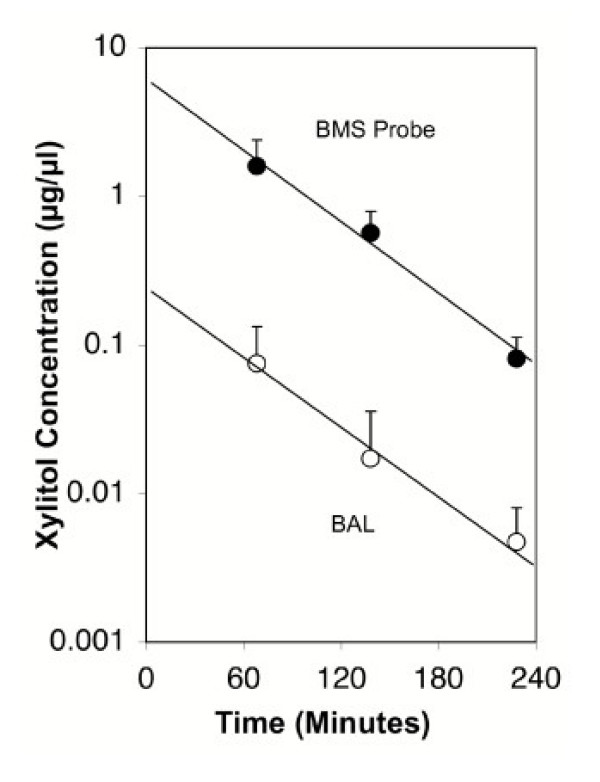
Semi-logarithmic plot of concentration of xylitol in human ASL plotted against time from the start of nebulization. The closed circles represent BMS probe data and open circles represent BAL data.

Xylitol concentration at various time points in the BMS probe and BAL is shown in Figure 3. Linear shape of the time-concentration graph using a semi-logarithmic model predicts a first-order kinetics. The concentration of xylitol in bronchial ASL collected by the BMS probe was at least one log higher than the BAL concentration after correcting for dilution.

**Figure 4:**
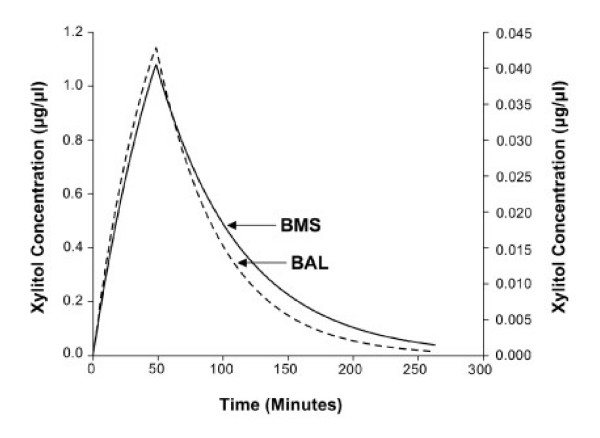
Simulated xylitol concentration versus time profile for the BMS probe and BAL methods. The solid line represents the simulated curve using population parameters (Table 1) obtained for the BMS probe method and the dashed line represents the simulated curve for the BAL method.

Figure 4 shows the plots for simulated xylitol concentration-time profile for BAL and BMS methods. The population estimates for the k, t_1/2_, R/V, and MRT values for the BAL and BMS methods are presented in Table 1. The half-life was 34.5 minutes for the BAL and 45.0 minutes for the BMS method. The MRT for the BMS method was 64.9 minutes, which is comparable to 49.8 minutes obtained for the BAL method.

**Table 1:** Summary of the population pharmacokinetic parameters of xylitol in ASL obtained by the BMS probe and BAL liquid.

	**BMS Probe**	**BAL**
**k (1/min)**	0.015	0.02
**t_1/2 _(min)**	45.0	34.5
**R/V (μg/μl-min)**	0.032	0.0014
**MRT (min)**	64.9	49.8

## Discussion

In this study, we evaluated the airway retention time of aerosolized xylitol using two methods. We previously reported the safety of aerosolized xylitol in normal volunteers. We now show that xylitol can be deposited in conducting airways after nebulization. After a single dose nebulization, xylitol was detected in ASL for at least 3 hours. This retention time is shorter than our *in vitro *data using the same detection method; however, the prolonged nebulization time (mean of 48 minutes) and the constant airway exposure during that time have to be considered when interpreting these data. Further, we found that the concentration was higher in the airways as sampled by the probe compared to alveoli plus airways as sampled by BAL.

ASL collection using a microsampling probe may prove valuable as a research tool and possibly aid in patient care. We found it safe and easy to use. BAL mostly samples alveolar fluid and, hence, significantly underrepresents concentration of products in the airway. The probe, in contrast, collects fluid directly from the airways without much dilution by alveolar liquid. A limitation of the probe is that it most likely collects volume of liquid in excess of the actual ASL by capillary action, drawing liquid from the mucosa and submucosa [16]. It may also stimulate submucosal gland secretion. Currently, however, there is no accurate method of ASL collection without altering its composition.

Not surprisingly, xylitol concentration was higher using BMS probe sampling compared to BAL, which is expected given the inhaled route of administration and specific sampling of ASL without any dilution by alveolar compartment. This is in contrast to the previous study using a microsampling probe and BAL, where Levaquin concentrations obtained from BAL were twice as high as that from the airway probe after oral dosing [9].

As to mechansims of clearance of xylitol from the airways, there are several possibilities, including mucociliary clearance, exhalation during tidal breathing, diffusion across the airway epithelia, and drug metabolism. Our data does not favor a particular mechanism. The airway retention time was significantly longer *in vitro *than *in vivo*. In our *in vitro *experiments, there are only airway epithelial cells without mucociliary clearance and relatively large volumes of xylitol were added to the apical surface of respiratory epithelim, which may have prolonged the retention time by adding to the distance of diffusion.

It was interesting to note that the ASL volume retrieved by the probes was higher after 3 hours compared to earlier time points. One possible explanation is the learning curve with the use of the microsampling probe to collect ASL. In this study, however, we recruited and completed the study on subjects assigned to the 3-hour time point before the 1.5-hour group. One of the possibilities for the increase in ASL volume at 3 hours is an osmotic effect of xylitol, resulting in dilution of ASL.

In the past, xylitol was used as an intravenous nutrition in doses as high as 0.25 gm/kg/hr [17]. After intravenous use, the half-life of xylitol is about 20 minutes in humans [18]. Exogenous xylitol is rapidly oxidized in the liver by NAD-linked polyol dehydrogenase into xylulose, which is then is phosphorylated and eventually metabolized by glycolysis or gluconeogenesis [19]. Therefore, to be effective in the lower respiratory tract, xylitol must be administered via aerosol route.

To our knowledge, this is the first study to assess airway deposition and retention time of aerosolized xylitol. A few limitations must be acknowledged. First, this study was done in healthy volunteers. In patients with lung disease, such as cystic fibrosis and those who are critically ill, the airway retention time may be different due to difference in epithelial integrity and permeability. Second, we did not study time points beyond 3 hours after nebulization. Third, because of the prolonged nebulization time and the preparation time for bronchoscopy, we were unable to assess the early pharmacokinetics of aerosolized xylitol. Fourth, we did not study clearance in proximal airway segments such as trachea or main stem bronchi. Finally, for safety and comfort reasons, ASL samples were collected from different sets of volunteers at different time points, which may have contributed to intersubject variability of the clearance estimates.

In conclusion, the MRT of aerosolized xylitol was greater than 1 hour. Aerosolized xylitol may be effective by transiently enhancing the innate immunity of the ASL and maintaining a sterile lung compartment, and, thus, prevent colonization in patients who are ventilated and in subjects with cystic fibrosis.

## Abbreviations

ASL: airway surface liquid

BAL: bronchoalveolar lavage

BMS: bronchoscopic microsampling

FEV: forced expiratory volume

NMR: nuclear magnetic resonance

MRT: mean residence time

## Competing interests

The author(s) declare that they have no competing interests.

## Authors' contributions

All authors read and approved the final manuscript.
